# External cephalic version outcomes with tocolysis and sedation: A 10‐year retrospective cohort study

**DOI:** 10.1002/ijgo.70711

**Published:** 2025-12-13

**Authors:** Javier Sánchez‐Romero, Rosa María Gallego‐Pozuelo, José Eliseo Blanco‐Carnero, Javier Herrera‐Giménez, Fernando Araico‐Rodríguez, Alberto Rafael Guijarro‐Campillo, Aníbal Nieto‐Díaz, Catalina de Paco‐Matallana

**Affiliations:** ^1^ Department of Obstetrics and Gynecology ‘Virgen de la Arrixaca’ University Hospital Murcia Spain; ^2^ Department of Obstetrics and Gynecology, Pediatrics and Surgery University of Murcia Murcia Spain; ^3^ Maternal‐Fetal Medicine, Reproduction and Gynecology Research Group Biomedical Research Institute of Murcia Pascual Parrilla–IMIB Murcia Spain

**Keywords:** breech presentation, cesarean section, external cephalic version, propofol, sedation, tocolysis

## Abstract

**Objective:**

To evaluate the effectiveness and safety of external cephalic version (ECV) performed with tocolysis and sedation or spinal anesthesia, and to identify predictors of ECV success, complications, and delivery outcomes after successful ECV.

**Methods:**

This 10‐year cohort study included 990 pregnant women with term non‐cephalic presentation at a tertiary hospital in Spain. Data were collected retrospectively (2014–2020) and prospectively (2020–2024). All ECVs followed a standardized protocol using ritodrine plus either propofol sedation or spinal anesthesia. Multivariable logistic regression identified predictors of ECV success and complications, and delivery outcomes were recorded.

**Results:**

ECV was successful in 691/990 (69.8%). Factors associated with higher success included a deepest amniotic fluid pocket >30 mm (OR = 1.80; 95%CI 1.04–3.11) and transverse lie (OR = 3.60; 95%CI 1.74–7.45). Lower success was observed in nulliparous women (OR = 0.55; 95%CI 0.40–0.76) and those with BMI between 30 and 35, and >35 kg/m^2^ (OR = 0.53 and 0.49 respectively). Complications occurred in 101/990 (10.2%), including 67/990 (6.7%) emergent cesarean sections. After successful ECV, 556/691 (80.5%) delivered vaginally.

**Conclusion:**

In this high‐volume tertiary center, ECV performed with systematic tocolysis and sedation or spinal anesthesia achieved high success and low complication rates, supporting the safety and feasibility of this optimized protocol.

## 1 INTRODUCTION

Non‐cephalic presentation occurs in approximately 3% of term pregnancies,^1^ presenting a clinical challenge for obstetricians. Management options include elective cesarean delivery, vaginal breech delivery, or external cephalic version (ECV).^2^

The publication of the Term Breech Trial (TBT) in 2000 significantly influenced clinical decision making. Initial findings suggested an increased risk of perinatal mortality with vaginal breech delivery,^3^ but subsequent follow‐up studies provided less consistent evidence.^4^ Nevertheless, the TBT led to a global rise in cesarean rates.^5^ In response, the World Health Organization (WHO) has promoted ECV as a strategy to reduce unnecessary cesarean rates.^6^

Despite the recognized benefits of ECV, its success rate remains variable. Several maternal and fetal factors, including multiparity, transverse lie, posterior placental location, black maternal race, and an amniotic fluid index greater than 10 cm, have been linked to higher success rates.^7^, ^8^, ^9^, ^10^ Additionally, various interventions, such as tocolytics (ritodrine,^11^ nifedipine,^11^ atosiban,^12^ nitroglycerin^13^) and anesthesia (propofol sedation, spinal anesthesia) have been explored to enhance ECV outcomes.^9^, ^10^, ^11^, ^12^, ^13^, ^14^, ^15^, ^16^

However, data are limited on the combined effect of tocolysis and sedation in ECV success and complications. Moreover, understanding the relationship between ECV success and subsequent delivery modes is essential for guiding clinical decisions.

The primary aim of this study was to evaluate the effectiveness and safety of a standardized external cephalic version protocol systematically combining tocolysis with deep sedation or spinal anesthesia in a high‐volume tertiary center. Secondary objectives included identifying predictors of ECV success and complications under this protocol, and description of subsequent delivery and neonatal outcomes. By addressing these aspects, the study seeks to contribute to optimizing management strategies for breech presentation at term.

## 2 MATERIALS AND METHODS

### 2.1 Study design

This single‐center cohort study was conducted at a tertiary care university hospital in Spain, between January 1, 2014, and December 31, 2024. It included a retrospective component (January 2014–December 2020) and a prospective component (January 2020–December 2024), during which the same standardized ECV protocol combining tocolysis with deep sedation or spinal anesthesia was applied. For the retrospective component (2014–2020), data were extracted from electronic medical records and standardized ECV procedure forms using the same predefined variables and definitions as in the prospective component, ensuring consistency in exposure, procedure, complication, and outcome recording across the study period.

Informed written consent was obtained from all prospectively recruited patients, and patient information was kept confidential; all participants gave explicit consent for the publication of anonymized clinical and outcome data in accordance with institutional and international ethical standards. The study received approval from the Institutional Review Board (2020‐5‐6‐HCUVA) and adhered to the ethical principles outlined in the 2013 Helsinki Declaration by the World Medical Association.

### 2.2 Participants

All eligible women undergoing ECV were included. During consultations, obstetrical history and ultrasound scans were reviewed to confirm fetal position, biometry, placental location, and amniotic fluid. ECV was offered at 36 weeks of pregnancy to pregnant women with a breech presentation or transverse/oblique lie and no contraindications for vaginal delivery. Women meeting any of the following exclusion criteria were deemed ineligible for the procedure: severe pre‐eclampsia, confirmed membrane rupture, anhydramnios (amniotic fluid pocket <15 mm), recent vaginal bleeding, or an absolute indication for cesarean delivery (i.e. placenta previa).

### 2.3 Procedure

The procedure followed a standardized protocol previously published.^17^ All participants fasted for a minimum of 8 hours before the procedure. All procedures were performed in an operating room within a dedicated ECV program by a multidisciplinary team including at least four senior obstetricians with extensive ECV experience, a midwife, and an anesthesiologist. Each ECV was carried out by a minimum of two senior obstetricians together with the midwife and anesthesiologist, with obstetrical residents participating under direct supervision.^18^

Pre‐procedure evaluations by the anesthesiologist included a thorough assessment of the patient's health. Ritodrine was administered at a dose of 0.2 mg/min for 30 minutes before the ECV. Continuous monitoring of vital signs, including temperature, noninvasive blood pressure, heart rate, electrocardiogram, and oxygen saturation, was conducted throughout the procedure.

Patients were positioned in a 15° Trendelenburg position. Anesthesia protocol was determined by anesthesiologist criteria with options including 1–1.5 mg/kg of propofol or spinal anesthesia consisting of 5 mg of bupivacaine and 20 μg of intrathecal fentanyl. Two ECV attempts were performed by the obstetricians using the forward‐roll technique. Fetal position was reassessed with an ultrasound immediately following the procedure, and fetal well‐being was monitored for 4 hours using a cardiotocograph. Rhesus‐negative patients received anti‐D immunoglobulin. On the day following the procedure, fetal well‐being was further ensured through 1‐hour cardiotocographic monitoring.

### 2.4 Outcomes

The procedure was deemed successful if cephalic presentation was achieved. Maternal and fetal complications occurring within 24 hours post‐procedure were documented. Major vaginal bleeding was defined as visible blood loss exceeding 50 mL or any episode raising suspicion of placental abruption, in accordance with the Royal College of Obstetricians and Gynecologists.^19^ Spotting referred to minor blood loss less than 50 mL. Uterine contractions were defined as regular and painful uterine activity occurring after ECV but without associated cervical change. Non‐reassuring fetal heart rate patterns were classified according to the criteria of the Spanish Society of Obstetrics and Gynecology (SEGO),^20^ which include acute prolonged decelerations and fetal bradycardia.

### 2.5 Statistical analysis

Statistical analyses were performed using Stata/BE version 18.0 (StataCorp, College Station, TX, USA). Normality and equal variance assumptions were assessed with the Shapiro–Wilk and Levene tests. Comparisons of continuous variables were conducted using Student *t*‐test or the Mann–Whitney *U* test, while proportions were compared using Pearson *χ* ^2^ test or Fisher exact test, as appropriate. Continuous outcomes were expressed as median with interquartile range, and binary outcomes were reported as count (frequency) or odds ratios (OR) with 95% confidence intervals (CI).

The primary outcome was ECV success, and the secondary outcome was the incidence of ECV‐related complications. Additional comparisons were performed for obstetric history, anthropometric variables, third‐trimester estimated fetal weight, placental location, and fetal presentation. Multivariable logistic regression was used to analyze both primary and secondary outcomes. Variables with a *P*‐value < 0.20 in the bivariate analysis, as well as variables selected a priori based on clinical relevance and previous evidence,^21^, ^22^ were entered simultaneously; no automated stepwise procedures were applied. Linearity in the logit for continuous predictors was assessed graphically. Collinearity was evaluated by fitting an ordinary least‐squares model with the same set of covariates and calculating variance inflation factors (VIF). All VIF values were <5, indicating no relevant multicollinearity. Results were expressed as odds ratios with corresponding 95% confidence intervals and *P*‐values. Mode of delivery after successful ECV was also reported. Statistical significance was set at *P* < 0.05, and all analyses were two‐tailed.

## 3 RESULTS

### 3.1 Participant characteristics

**FIGURE 1:**
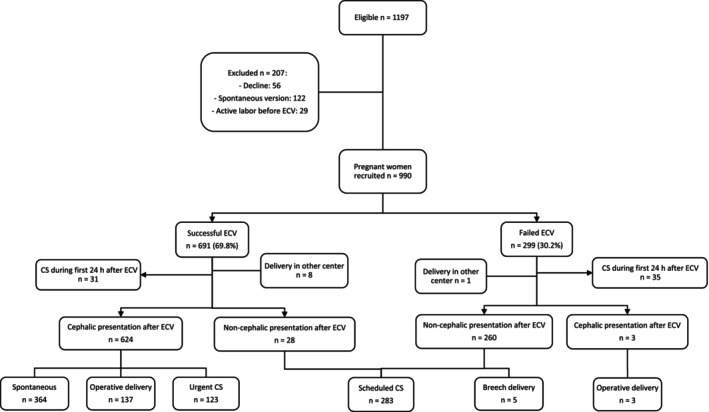
Flowchart of pregnant women included in the analysis and the external cephalic version outcome and mode of delivery.

During the study period, ECV was offered to 1197 pregnant women, with 56 (4.7%) declining the procedure (Figure 1). Spontaneous cephalic presentation before ECV occurred in 122 cases (10.2%), and 29 women (2.4%) began active labor before undergoing ECV. Descriptive comparisons between the retrospective and prospective cohorts are provided in Table S1.

Baseline characteristics are summarized in Table 1. Both groups were generally comparable, except for differences in maternal age (33.4 versus 32.5 years; *P* = 0.002), nulliparity (50.4% versus 68.9%; *P* < 0.001), body mass index (BMI; calculated as weight in kilograms divided by the square of height in meters) (25.8 versus 26.6; *P* < 0.001), amniotic fluid pocket (51.2 versus 43.5 mm; *P* < 0.001), and transverse lie (11.7% versus 3.3%; *P* < 0.001).

**TABLE 1:** Baseline characteristics.

Variable	Overall, *n* = 900	Successful ECV, *n* = 691 (69.8%)	Failed ECV, *n* = 299 (30.2%)	*p*
Age, years	33.2 (6.8)	33.4 (6.8)	32.5 (6.5)	**0.002**
Nulliparous	554 (56.0%)	348 (50.4%)	206 (68.9%)	**<0.001**
Previous cesarean section	47 (4.7%)	35 (5.1%)	12 (4.0%)	0.475
BMI, kg/m^2^	26.0 (6.2)	25.8 (6.0)	26.6 (6.6)	**<0.001**
<25 kg/m^2^	407 (41.1%)	300 (43.6%)	107 (36.2%)	**0.028**
25–30 kg/m^2^	407 (41.4%)	252 (36.3%)	107 (36.2%)	
30–35 kg/m^2^	359 (36.5%)	98 (14.2%)	56 (18.9%)	
>35–40 kg/m^2^	64 (6.5%)	38 (5.5%)	26 (8.8%)	
Maternal comorbidity				0.163
GDM	47 (4.8%)	37 (5.4%)	10 (3.4%)	
Pregestational DM	13 (1.3%)	12 (1.8%)	1 (0.3%)	
Cholestasis	2 (0.2%)	2 (0.3%)	0 (0)	
Gestational hypertension	4 (0.4%)	3 (0.4%)	1 (0.3%)	
Preeclampsia	8 (0.8%)	6 (0.8%)	2 (0.7%)	
Others	6 (0.6%)	6 (0.9%)	0 (0)	
Gestational age, weeks	37.4 (0.6)	37.3 (0.6)	37.4 (0.6)	0.436
Estimated fetal weight, g	2,762 (510)	2,765 (540)	2745 (501)	0.066
Placental location				0.554
Anterior	564 (57.0%)	387 (56.0%)	177 (59.2%)	
Posterior	359 (36.5%)	249 (36.0%)	95 (31.8%)	
Fundal	24 (2.4%)	15 (2.2%)	9 (3.0%)	
Lateral wall	58 (5.9%)	40 (5.8%)	18 (6.0%)	
AF pocket, mm	49.0 (20.9)	51.2 (22.5)	43.5 (16.1)	**<0.001**
<30 mm	61 (7.4%)	34 (6.1%)	27 (10.2%)	**0.035**
>30 mm	763 (92.6%)	525 (93.9%)	238 (89.8%)	
Fetal position				**<0.001**
Breech	899 (90.8%)	610 (88.3%)	289 (96.7%)	
Transverse lie	91 (9.2%)	81 (11.7%)	10 (3.3%)	
Analgesia				0.423
Deep sedation	945 (95.5%)	662 (95.8%)	283 (94.7%)	
Spinal analgesia	45 (4.6%)	29 (4.2%)	16 (5.4%)	

*Note*: Continuous variables are summarized as median (IQR). Categorical variables are summarized as count (frequency).

Abbreviations: AF, amniotic fluid; DM, diabetes mellitus; ECV, external cephalic version; GDM, gestational diabetes mellitus.

Maternal comorbidities are presented in Table 2. These conditions affected 80 participants and were comparable between groups (*P* = 0.163). Rare comorbidities included Chagas disease, maternal HIV infection with undetectable viral load, maternal hepatitis B, Arnold‐Chiari type 2 syndrome, mitochondrial myopathy, and Crohn disease, reported in six women.

**TABLE 2:** External cephalic version complications.

ECV complications	Successful ECV, *n* = 691	Failed ECV, *N* = 299	*p*
	Count (frequency)	Count (frequency)	<0.001
Non‐reassuring FHR	20 (2.9%)	19 (6.4%)	
Major vaginal bleeding	12 (1.8%)	9 (3.0%)	
Spotting	11 (1.6%)	8 (2.7%)	
Uterine contractions	1 (0.1%)	12 (4.0%)	
PROM	5 (0.7%)	0 (0)	
Cord prolapse	3 (0.4%)	0 (0)	
Bronchoaspiration	0 (0)	1 (0.3%)	

Abbreviations: ECV, external cephalic version; FHR, fetal heart rate; PROM, premature rupture of membranes.

### 3.2 ECV success rate

**FIGURE 2:**
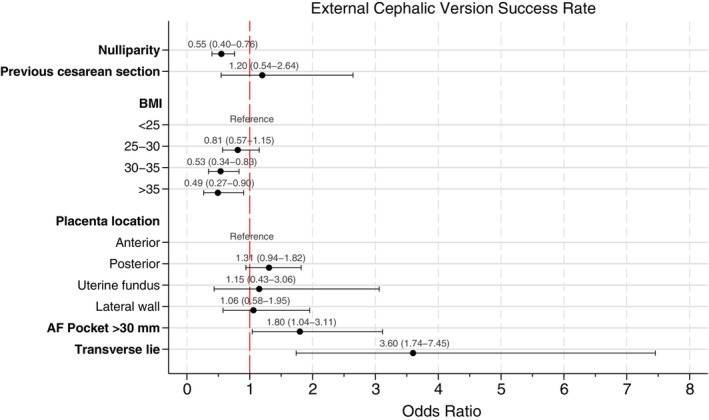
Multivariate analysis of external cephalic version success rate. Multivariate analysis was adjusted by maternal age, nulliparity, previous cesarean section, body mass index (BMI), placental location, amniotic fluid pocket, and fetal position.

The ECV procedure was successful in 691 cases (69.8%, 95% CI 66.9%–72.6%). ECV success was higher in transverse lie (89.0%; 95% CI 82.6%–95.4%) compared with breech presentation (67.8%; 95% CI 64.8%–70.9%; *P* < 0.001). Multivariate analysis (Figure 2) identified maternal age (adjusted OR [aOR] 1.03; 95% CI 1.00–1.06; *P* = 0.025), amniotic fluid pocket greater than 30 mm (aOR 1.80; 95% CI 1.04–3.11; *P* = 0.036), and transverse lie (aOR 3.60; 95% CI 1.74–7.45; *P* = 0.001) as factors associated with increased success rates. Conversely, nulliparity (aOR 0.55; 95% CI 0.40–0.76; *P* < 0.001), BMI between 30 and 35 (aOR 0.53; 95% CI 0.34–0.83; *P* < 0.005), and BMI greater than 35 (aOR 0.49; 95% CI 0.27–0.90; *P* < 0.022) were associated with reduced success rates. Previous cesarean delivery showed no significant association with ECV success (aOR 1.20; 95% CI 0.54–2.64; *P* = 0.651). The area under ROC curve for this predicting model was 66.4%.

### 3.3 ECV complications

**FIGURE 3:**
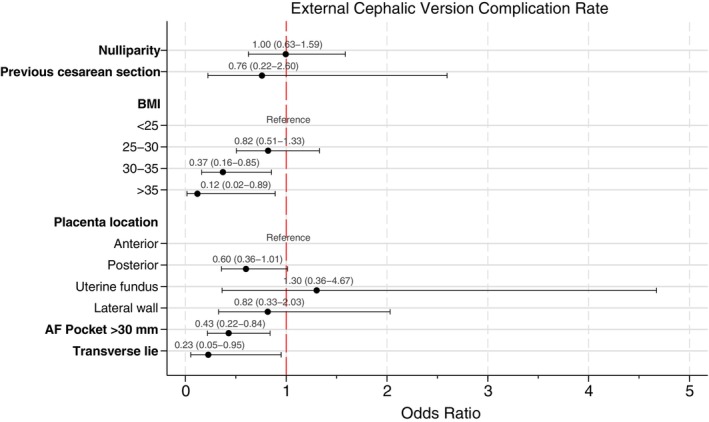
Multivariate analysis of external cephalic version complication rate. Multivariate analysis was adjusted by nulliparity, previous cesarean section, body mass index (BMI), placental location, amniotic fluid pocket, and fetal position.

During the first 24 hours post‐ECV, 101 complications (10.2%) were documented (Table 3). Between these, 66 women (6.7%) requiring emergent cesarean delivery. Multivariate analysis (Figure 3) revealed associations between complications and BMI between 30 and 35 (aOR 0.37; 95% CI 0.16–0.85; *P* = 0.020), BMI greater than 35 (aOR 0.12; 95% CI 0.02–0.89; *P* = 0.038), amniotic fluid pocket greater than 30 mm (aOR 0.43; 95% CI 0.22–0.84; *P* = 0.014), and transverse lie (aOR 0.23; 95% CI 0.05–0.95; *P* = 0.042). Nulliparity (*P* = 0.986) and previous cesarean (*P* = 0.662) were not associated with increased complications. The area under ROC curve for this predicting model was 66.3%.

**TABLE 3:** Labour onset, mode of delivery and neonatal outcomes after an ECV between patients with vaginal delivery attempt and patients who required cesarean section during first 24 h after ECV.

Variable	Vaginal delivery attempt after ECV (*n* = 632)	CS during first 24 h after ECV (*n* = 66)	*p*
Gestational age at delivery, weeks	40.0 (1.9)	37.3 (0.7)	0.146
Labour onset			
Spontaneous	343 (54.3%)		
Induced	289 (45.7%)		
Mode of delivery			
Spontaneous delivery rate	369 (58.4%)		
Operative delivery rate	140 (22.2%)		
Cesarean delivery rate	123 (19.5%)	66 (100%)	
Neonatal weight, g	3283 (610)	2833 (325)	**<0.001**
Arterial cord pH<7	3 (0.6%)	0 (0%)	0.561
1‐min Apgar score <7	21 (3.3%)	21 (31.8%)	**<0.001**
5‐min Apgar score <7	4 (0.6%)	2 (3.0%)	0.104
Neonatal admission	13 (2.1%)	9 (13.6%)	**<0.001**
nICU admission	6 (0.9%)	2 (3.0%)	0.144
Neonatal death	1 (0.1%)	0 (0%)	0.484

*Note*: Continuous variables are summarized as median (IQR). Categorical variables are summarized as count (frequency).

Abbreviations: ECV, external cephalic version; nICU, neonatal Intensive Care Unit.

Just one patient experienced bronchoaspiration immediately following ECV, requiring admission to the maternal care unit for antibiotic treatment. Despite achieving cephalic presentation, a cesarean delivery was performed 7 days later due to the incident. The neonate's Apgar score was 9/10, with an umbilical vein pH of 7.32. Both mother and child were discharged without sequelae.

### 3.4 Mode of delivery and neonatal outcome after ECV

Nine women (0.91%) delivered at external centers. Of the 981 women who delivered at the study center, 66 (6.7%) underwent cesarean delivery within the first 24 hours post‐procedure. Among these cases, nine neonates (13.6%) required neonatal admission due to complications such as transient tachypnea (three cases), jaundice (two cases), respiratory distress syndrome (two cases), hypoglycemia, and hypotonia. Two neonates required neonatal intensive care unit (NICU) admission for transient tachypnea and respiratory distress syndrome, respectively. All neonates were discharged without sequelae. Among women with successful ECV, 28 (4.1%) experienced spontaneous reversion to a non‐cephalic presentation at delivery. Conversely, three women (1.0%) with an unsuccessful ECV subsequently delivered in cephalic presentation.

A total of 632 women (63.8%) attempted vaginal delivery after successful ECV, with 509 (80.5%) achieving vaginal births (Figure 1). Neonatal results, including onset of labor and mode of delivery, are detailed in Table 3. Thirteen neonates (2.1%) required neonatal admission, and six (0.9%) required NICU admission for reasons such as maternal postpartum hemorrhage, neonatal respiratory distress syndrome, transient tachypnea, hypoglycemia, jaundice, subgaleal hematoma, and chorioamnionitis. Four neonates were admitted due to congenital conditions, including CHARGE syndrome, Loucks‐Innes syndrome, propionic acidemia, and Zellweger syndrome.

Neonatal weight (*P* < 0.001), Apgar score at 1 minute less than 7 (*P* < 0.001), and neonatal admission (*P* < 0.001) differed significantly between women who attempted vaginal delivery and those who required an emergency cesarean within the first 24 hours after ECV (Table 3).

One neonatal death (0.2%) and two stillbirths (0.2%) were recorded. The neonatal death involved a newborn prenatally diagnosed with Loucks‐Innes syndrome and tetralogy of Fallot, who succumbed to sepsis after 60 days of NICU admission. One stillbirth occurred following premature rupture of membranes and subsequent cord prolapse 12 days post‐ECV. The other was attributed to an intrauterine death of unknown cause 7 days after a failed ECV attempt (Table 3).

## 4 DISCUSSION

This study provides a comprehensive 10‐year overview of ECV practice, reporting outcomes from over 990 consecutive attempts. It includes detailed data on eligibility criteria, refusal and reversion rates, and maternal and neonatal complications, offering a robust insight into the safety and efficacy of ECV in a high‐volume center.

The observed ECV success rate in our cohort of 69.8% is notably higher than those described in previous studies, which range from 44.3% to 58.4%.^7^, ^16^, ^22^ This difference is likely attributable to key aspects of our setting: (1) the use of a standardized protocol with systematic tocolysis and adjuvant deep sedation or spinal anesthesia, and (2) the performance of the procedure by an experienced, dedicated team. The combination of tocolysis with either spinal anesthesia or propofol sedation may facilitate fetal repositioning by promoting uterine relaxation,^23^ reducing maternal discomfort,^15^ and minimizing sympathetic activity,^15^ which is consistent with the higher success rates observed in our cohort.

All ECV procedures were performed by a consistent team of two senior obstetricians with over 10 years of experience and more than 900 ECV procedures performed during the study period. No residents participated directly in the procedures. All interventions occurred in an operating room setting to allow immediate cesarean delivery if needed. The operator experience is associated with an increase in the ECV success rate, mainly in nulliparae.^24^, ^25^ Other authors proposed 130 procedures in nulliparae to obtain a 70% of success rate.^26^

To our knowledge, this is the largest known cohort evaluating ECV outcomes with consistent use of tocolysis and either deep sedation or spinal anesthesia. Our findings regarding the association of BMI, amniotic fluid volume, and placental location with ECV success are consistent with previous reports,^7^, ^12^, ^27^, ^28^ but are here confirmed in the specific context of a standardized protocol with systematic tocolysis and anesthesia support, providing a more precise estimate of their impact under an optimized strategy.

Regarding maternal complications, we provided a comprehensive analysis of all adverse events, reporting an overall complication rate of 10.2% and an emergency cesarean rate of 6.7%. Other studies have reported lower complication rates (5.3%–6.6%), but these often lacked the use of tocolysis or anesthesia and correspondingly reported lower success rates.^7^, ^29^ When spinal anesthesia and tocolysis are used, higher success rates (up to 83%) have been observed but with an elevated cesarean rate (14.3%).^30^ Despite reporting a case of maternal bronchoaspiration, this was the only severe maternal adverse event, emphasizing the importance of rigorous pre‐anesthetic evaluation and adherence to fasting guidelines,^31^ as delayed gastric emptying is common during pregnancy.^32^

Regarding neonatal complications that arose after complicated procedures, we reported a neonatal admission rate of 13.6% (*n* = 9). However, the Apgar score at 5 minutes of life (*P* = 0.104) and arterial blood cord pH less than 7 (*P* = 0.104) did not differ between ECV that required cesarean delivery during the first 24 hours after procedure and those with vaginal delivery attempt. Serious neonatal complications, including stillbirth, neonatal death, low Apgar scores, low umbilical artery pH, and need for therapeutic hypothermia, remain rare and occur at similar rates in ECV and non‐ECV groups.^33^, ^34^

Regarding mode of delivery after ECV, the findings further demonstrate a high vaginal delivery rate (80.5%) after successful ECV, with spontaneous delivery accounting for 58.4% and operative delivery for 22.2%. This aligns with results from meta‐analyses, such as those by De Hund et al.,^35^ who reported a vaginal delivery rate of 79.3%. However, centers with established breech delivery protocols have reported rates as high as 85.3%.^36^ These results support ECV as a highly effective intervention for reducing cesarean delivery due to malpresentation.

Our results support offering ECV with appropriate tocolysis and spinal anesthesia or sedation with propofol as part of routine clinical care for term breech presentations, provided it is conducted by experienced teams in adequately equipped settings. A successful ECV resulted in a high rate of vaginal deliveries, underlining the potential of ECV in reducing cesarean deliveries.

This observational study carries inherent limitations. First, the use of two different anesthetic modalities without randomized allocation may have introduced selection bias and confounding by indication. Second, despite all procedures being performed within a dedicated ECV program by senior obstetricians, anesthesiologists, and a specialized midwife, the choice of anesthetic technique ultimately depended on the attending anesthesiologist and specific clinical scenarios. Third, maternal BMI was only assessed in the first trimester, not accounting for changes during pregnancy. Fourth, the retrospective component may have led to underreporting of minor complications. Finally, our findings derive from a high‐volume tertiary center with a structured ECV team, and may not be directly generalizable to low‐volume units or settings without similar expertise and anesthetic resources; external validation in different contexts is warranted. This question, particularly regarding the anesthetic strategy, is being further addressed in our ongoing randomized controlled trial (PropoSpinECV—ClinicalTrials ID: NCT06449430).^37^

The primary strength of this study lies in its longitudinal design and detailed follow up, enabling robust analysis of maternal, fetal, and procedural variables. To our knowledge, it is the most comprehensive dataset examining ECV success and complication predictors using sedation‐based protocols. Our protocol systematically integrated tocolysis with either spinal anesthesia or sedation, in accordance with emerging recommendations from several national and international societies.^2^

In conclusion, external cephalic version is a safe and effective intervention for reducing cesarean deliveries in cases of non‐cephalic presentation at term. When performed under optimal conditions, including the use of tocolysis and sedation and by experienced clinicians, ECV achieves high success rates with low complication rates. It should be routinely offered to eligible women wishing to pursue vaginal delivery, as part of evidence‐based obstetrical care.

## AUTHOR CONTRIBUTIONS

Data compilation was conducted by JSR and RMGP. Statistical analysis was executed by JSR, ARGJ, and CdPM. All the procedures were performed by the ECV super‐specialized team. JEBC, JHG, and FAR are part of this team. Technical and academic guidance were generously provided by JEBC, AND, and JSR. All the authors participated in drafting and redaction of the manuscript and approved the final version.

## FUNDING INFORMATION

This institution has covered the expenses for open access publication.

## CONFLICT OF INTEREST STATEMENT

Javier Sánchez‐Romero, Rosa María Gallego‐Pozuelo, José Eliseo Blanco‐Carnero, Catalina de Paco‐Matallana and Aníbal Nieto‐Diaz are affiliated with the University of Murcia.

## Supporting information


**Table S1.** Baseline and outcome characteristics in the retrospective versus prospective cohorts.

## Data Availability

The data that support the findings of this study are available from the corresponding author upon reasonable request.
