# Supplementary material for: External cephalic version outcomes with tocolysis and sedation: A 10‐year retrospective cohort study
Source: Int J Gynaecol Obstet. 2025 Dec 13;173(3):1343–51. doi: 10.1002/ijgo.70711 (PMC13173633; doi:10.1002/ijgo.70711)
Supplement: Supplementary file 1 — Table S1. Baseline and outcome characteristics in the retrospective versus prospective cohorts. [file IJGO-173-1343-s001.docx]

**Supplementary Table S1**. Baseline and outcome characteristics in the retrospective vs prospective cohort. Continuous variables are summarized as median (interquartile range). Categorical variables are summarized as count (percentage). ECV: External cephalic version. CS: Cesarean section. BMI: Body mass index. GDM: Gestational diabetes mellitus. DM: Diabetes mellitus. AF: Amniotic fluid. PROM: Preterm rupture of membranes. FHR: Fetal heart rate. ICU: Intensive care unit.

|  | **Total**  **n = 990** | **Retrospective**  **n = 645** | **Prospective**  **n = 345** | ***p*** |
| --- | --- | --- | --- | --- |
| **Maternal age, years** | 33.2 (6.79) | 33.0 (6.85) | 33.5 (6.54) | 0.097 |
| **Gestational age at ECV, weeks** | 37.4 (0.57) | 37.3 (0.57) | 37.4 (0.57) | <0.001 |
| **Nulliparity** | 554 (56%) | 362 (56.1%) | 192 (55.7%) | 0.887 |
| **Previous CS** | 47 (4.6%) | 33 (5.1%) | 14 (4.1%) | 0.456 |
| **BMI, Kg/m^2^** | 26 (6.2) | 26 (6.4) | 26 (5.72) | 0.764 |
| < 25 | 407 (41.4%) | 263 (41%) | 144 (42%) | 0.846 |
| 25 - 30 | 359 (36.5%) | 233 (36.3%) | 126 (36.7%) |  |
| 30 - 35 | 154 (15.7%) | 100 (15.6%) | 54 (15.7%) |  |
| > 35 | 64 (6.5%) | 45 (7.02%) | 19 (5.54%) |  |
| **Maternal comorbidity** |  |  |  | <0.001 |
| GDM | 47 (4.79%) | 13 (2.04%) | 34 (9.86%) |  |
| Pregestational DM | 13 (1.32%) | 10 (1.57%) | 3 (.87%) |  |
| Cholestasis | 2 (0.2%) | 1 (0.2%) | 1 (0.3%) |  |
| Gestational Hypertension | 4 (0.4%) | 4 (0.6%) | 0 (0%) |  |
| Preeclampsia | 8 (0.8%) | 3 (0.5%) | 5 (1.5%) |  |
| Others | 6 (0.6%) | 5 (0.8%) | 1 (0.3%) |  |
| **Placenta location** |  |  |  | <0.001 |
| Anterior | 564 (57%) | 391 (60.6%) | 173 (50.1%) |  |
| Posterior | 344 (34.7%) | 214 (33.2%) | 130 (37.7%) |  |
| Uterine fundus | 24 (2.4%) | 17 (2.6%) | 7 (2.0%) |  |
| Lateral wall | 58 (5.9%) | 23 (3.6%) | 35 (10.1%) |  |
| **AF Pocket, mm** | 49 (20.9) | 49.2 (20.7) | 48 (21.4) | 0.770 |
| < 30 mm | 61 (7.4%) | 35 (7.3%) | 26 (7.5%) | 0.901 |
| > 30 mm | 763 (92.6%) | 444 (92.7%) | 319 (92.5%) |  |
| **Estimated Fetal Weight, g** | 2762 (510) | 2774 (474) | 2724 (576) | 0.025 |
| **Transverse lie** | 91 (9.2%) | 56 (8.7%) | 35 (10.1%) | 0.448 |
| **Analgesia** |  |  |  | <0.001 |
| Sedation | 945 (95.5%) | 634 (98.3%) | 311 (90.1%) |  |
| Spinal anesthesia | 45 (4.6%) | 11 (1.7%) | 34 (9.9%) |  |
| **ECV success rate** | 691 (69.8%) | 472 (73.2%) | 219 (63.5%) | 0.002 |
| **ECV complication rate** | 101 (10.2%) | 71 (11%) | 30 (8.7%) | 0.249 |
| **ECV Complication** |  |  |  | 0.800 |
| PROM | 5 (5.0%) | 4 (5.6%) | 1 (3.3%) |  |
| Uterine contractions | 13 (12.9%) | 10 (14.1%) | 3 (10%) |  |
| Cord prolapse | 3 (3.0%) | 2 (2.8%) | 1 (3.3%) |  |
| Spotting | 19 (18.8%) | 15 (21.1%) | 4 (13.3%) |  |
| Major vaginal bleeding | 21 (20.8%) | 15 (21.1%) | 6 (20%) |  |
| Non-reassuring FHR | 39 (38.6%) | 24 (33.8%) | 15 (50.0%) |  |
| Bronchoaspiration | 1 (1.0%) | 1 (1.4%) | 0 (0%) |  |
| **Labour Onset** |  |  |  | <0.001 |
| Spontaneous | 343 (35.0%) | 254 (39.9%) | 89 (25.9%) |  |
| Induced | 289 (29.5%) | 172 (27.0%) | 117 (34.0%) |  |
| No labour onset | 349 (35.6%) | 211 (33.1%) | 138 (40.1%) |  |
| **Type of delivery** |  |  |  | <0.001 |
| Spontaneous | 369 (37.6%) | 245 (38.5%) | 124 (36%) |  |
| Operative | 140 (14.3%) | 109 (17.1%) | 31 (9.01%) |  |
| Urgent CS | 123 (12.5%) | 72 (11.3%) | 51 (14.8%) |  |
| Scheduled CS | 283 (28.8%) | 165 (25.9%) | 118 (34.3%) |  |
| Emergent CS 24 h after ECV | 66 (6.7%) | 46 (7.2%) | 20 (5.8%) |  |
| **Gestational age at delivery, weeks** | 39.4 (1.9) | 39.4 (2) | 39.3 (1.7) | 0.201 |
| **Newborn weight, g** | 3183 (590) | 3210 (575) | 3150 (615) | 0.431 |
| **pH** | 7.29 (0.1) | 7.29 (0.1) | 7.29 (0.1) | 0.888 |
| **pH below 7** | 5 (0.6%) | 5 (1.0%) | 0 (0%) | 0.079 |
| **Apgar 1** | 9 (0) | 9 (0) | 9 (0) | 0.059 |
| **Apgar 1 below 7** | 55 (5.6%) | 42 (6.6%) | 13 (3.8%) | 0.070 |
| **Apgar 5** | 10 (0) | 10 (0) | 10 (0) | 0.088 |
| **Apgar 5 below 7** | 11 (1.1%) | 9 (1.4%) | 2 (0.6%) | 0.241 |
| **Neonatal care admission** | 29 (3.0%) | 20 (3.1%) | 9 (2.6%) | 0.641 |
| **Neonatal ICU care admission** | 9 (0.9%) | 5 (0.8%) | 4 (1.2%) | 0.555 |
| **Neonatal death** | 3 (0.3%) | 1 (0.2%) | 2 (0.6%) | 0.251 |
